# Role of reactive thrombocytosis after primary cytoreductive surgery in advanced ovarian cancer

**DOI:** 10.3389/fonc.2022.926878

**Published:** 2022-09-08

**Authors:** Myeong-Seon Kim, Seung Hun Baek, Joseph J. Noh, Jung In Shim, Jun Hyeok Kang, Soo Young Jeong, Chel Hun Choi, Tae-Joong Kim, Jeong-Won Lee, Yoo-Young Lee

**Affiliations:** ^1^ Division of Gynecologic Oncology, Department of Obstetrics and Gynecology, St. Vincent’s Hospital, College of Medicine, The Catholic University of Korea, Seoul, South Korea; ^2^ Division of Gynecologic Oncology, Department of Obstetrics and Gynecology, Samsung Medical Center, Sungkyunkwan University School of Medicine, Seoul, South Korea

**Keywords:** epithelial ovarian cancer, primary cytoreductive surgery, reactive thrombocytosis, splenectomy, adjuvant chemotherapy

## Abstract

We investigated the incidence of reactive thrombocytosis after maximal cytoreductive surgery in advanced epithelial ovarian cancer (EOC) and its role in patient survival. We retrospectively reviewed the electronic medical records of patients who underwent primary cytoreductive surgery for advanced EOC from 1 January 2012 to 31 December 2017. We analyzed the serum platelet counts at various time points from before surgery, during the peri-operative period, and after each cycle of adjuvant chemotherapy. A total of 474 patients were eligible for the analysis. Among them, 401 patients (84.6%) had FIGO stage III disease status. The most common histology type was serous adenocarcinoma (405 patients, 85.4%). Seventy-nine patients (22.6%) received splenectomy, and optimal cytoreduction was achieved in 326 patients (68.8%). A week after surgery, thrombocytosis was observed in 165 patients (34.8%) in the entire cohort. Higher platelet counts were observed in patients with splenectomy compared with patients without splenectomy. In particular, thrombocytosis on the fifth cycle of adjuvant chemotherapy showed the most significant effects on overall survival in multivariate analysis. In a logistic regression model, splenectomy was significantly attributed to thrombocytosis on the fifth cycle of chemotherapy. Reactive thrombocytosis after primary cytoreductive surgery is associated with poor survival in advanced EOC, particularly when thrombocytosis is observed during adjuvant chemotherapy.

## Introduction

Ovarian cancer is the most lethal disease of the female genital tract. With relatively stable incidence overtime in most countries, it was estimated that there would be 295,414 new cases and 184,799 deaths worldwide in 2018 (1, 2). Ovarian cancer accounts for 2.5% of all cancer patients among females but results in 5% of all cancer deaths because of its high fatality rate (3). Four of five patients are diagnosed with advanced disease, contributing to their poor prognosis (4).

Maximal cytoreductive surgery and platinum-based combination chemotherapy are the mainstays of treatment for advanced epithelial ovarian cancer (EOC). Optimal cytoreduction is highly recommended to leave a small volume of disease, ideally no gross residual, to increase survival, and extra-uterine procedures, including resections of the bowels, diaphragm, or spleen, are frequently performed to achieve this goal (5).

Thrombocytosis after surgery for various diseases was observed in patients who underwent surgery on the bowel (6), bladder (7, 8), or spleen (9, 10). Concerns have been expressed for many years that thrombocytosis may decrease oncological outcomes. However, reports so far have evaluated the role of preoperative platelet counts on survival in the bladder (8), breast (11), lung (12), gastric (13), and colorectal (14) cancers only. In gynecologic malignancies, thrombocytosis as a paraneoplastic syndrome in ovarian cancer, in which the incidence was reported to range from 22.4% to 62.5% (15), was associated with advanced-stage disease, vascular thromboembolic complications, higher preoperative levels of CA-125, and a significantly shorter median time to disease progression (16). In one study, the use of an antiplatelet antibody to decrease platelet counts in a tumor-bearing mouse model significantly reduced tumor growth and angiogenesis, suggesting antiplatelet therapy as a new treatment strategy, especially in ovarian cancer patients with thrombocytosis during or after standard treatment. However, it has remained unclear whether reactive thrombocytosis after surgery is associated with poor survival, particularly in ovarian cancer, although this has been demonstrated in several types of solid malignancies (17–20). In this study, we investigated the incidence of reactive thrombocytosis after primary cytoreductive surgery and its association with survival in advanced EOC patients.

## Methods

### Ethical issues

The study was conducted according to the guidelines of the Declaration of Helsinki. It was approved by the Institutional Review Board (IRB) (No. 2020-03-141-001) of the Samsung Medical Center. This retrospective study did not require a tissue sample collection from the patients. The records of surgery and test results that had already been collected were analyzed. Even without the consent of the subject, there is no risk to the subject due to this study. For the above reasons, the informed consent of the patient was waived.

### Intervention

Patients were selected with the following inclusion criteria: patients (1) who underwent primary cytoreductive surgery at the Samsung Medical Center from 1 January 2012 to 31 December 2017; (2) those who were diagnosed with epithelial ovarian, fallopian, or peritoneal cancer (described as EOC); and (3) those who were diagnosed with advanced stage (FIGO stage III or IV). Patients who were treated with neoadjuvant chemotherapy (NAC), diagnosed with non-epithelial histology, early stage (FIFO stages I–II), or hematologic disease (e.g., idiopathic thrombocytosis, idiopathic thrombocytopenic purpura, and etc.) were excluded. Among 674 patients diagnosed with advanced ovarian cancer during the study period, 125 patients who had NAC, 55 patients without maximal cytoreductive surgery, and 22 patients who had non-epithelial ovarian cancer on final pathology were excluded, which resulted in 474 patients for final analysis.

For patients newly diagnosed with advanced EOC, platinum-based combination chemotherapy, mainly tri-weekly intravenous paclitaxel plus carboplatin, was followed for six cycles after primary cytoreductive surgery. Routine prophylactic anticoagulation management was performed in all patients, and vaccination to decrease the risk of postsplenectomy sepsis was given to patients who underwent splenectomy at the time of primary cytoreductive surgery.

Platelet counts were measured as part of the results of a complete blood count (CBC), which is routinely done for all patients within one month of primary cytoreductive surgery. It was also measured on every other day after surgery (post-operative day (POD) 1, POD 3, POD 5, POD 7, and so on) and one day before each cycle of adjuvant chemotherapy. In the case of multiple CBC results due to transfusion at any time point, platelet counts from CBC before transfusion were used for analysis. Overall, we were able to obtain at least 10 serial platelet counts over 6 months during primary treatment for these patients. Besides serum platelet counts, clinical variables such as age, pre-operative CA-125, FIGO stage, histology, and level of residual disease (complete gross resection = R0, gross residual disease less than 1 cm = R1, gross residual disease equal to or more than 1 cm = R2) after primary cytoreductive surgery were collected retrospectively. Optimal cytoreduction was defined as residuals less than 1 cm (R0 + R1).

We defined thrombocytosis as platelet counts the same or greater than 4.0 × 10^5^/mm^3^ based on previous studies (11, 12, 20, 21). Among the time points after surgery, we sought to find the best timing when thrombocytosis was associated with overall survival (OS). Then, thrombocytosis at this time point was analyzed with other variables in a Cox*-*regression model. Progression-free survival (PFS) was defined as the time from surgery to the first recurrence or last follow-up. OS was defined as the time from surgery to the date of death or last follow-up.

All statistical analyses were performed using IBM SPSS statistics software Version 25.0 (IBM Corp. Armonk, New York, USA). For the analysis of the distribution of data characteristics, median (range) or mean (standard deviation) were used to describe continuous variables. Categorical variables are shown as frequency (percentage). After confirmation of normal distributions with the Shapiro–Wilk test, the Mann–Whitney test was performed to compare median values, and the Student t-test was used to compare mean values. A Fisher’s exact test or chi-square test was used to compare categorical variables. For the analysis of survival outcomes, the Kaplan–Meier method with the log-rank test was used. For multivariate analysis, the Cox proportional hazard model with backward selection was used. Binary logistic regression analysis was used to identify attributable factors for reactive thrombocytosis after surgery. A *p*-value of <0.05 was considered statistically significant.

## Results

A total of 474 patients were eligible for this study. Characteristics of the patients are presented in **Table 1**. The median age was 54 (18–88) years old and the pre-operative CA-125 was 617 (6–16,719) IU/ml. Approximately 84.6% (401/474) of patients were FIGO stage III and 85.4% (405/474) had serous histology. The rates of R0, R1, and R2 were 46.0% (218/474), 22.8% (108/474), and 31.2% (148/474) respectively, meaning that optimal cytoreduction was achieved in 68.8% (326/474) of patients after primary cytoreductive surgery. The median interval between surgery and the first cycle of chemotherapy was 11 (6–61) days. Because splenectomy is associated with reactive thrombocytosis after surgery (9, 10), we divided the entire cohort into two groups based on splenectomy during cytoreductive surgery. Compared with patients without splenectomy, patients with splenectomy (22.6%, 79/474) showed higher levels of preoperative CA-125 (634 IU/ml [95% CI 6–15,241] *vs*. 859 IU/ml [95% CI 16–16,719], *p*-value = 0.035), a higher proportion of FIGO stage IV (25.3%, 20/79 *vs*. 13.4%, 53/395, *p*-value = 0.007) and a longer delay from surgery to the first cycle of chemotherapy (12 days [95% CI 7–61] *vs*. 11 days [6–55], *p*-value = 0.003). However, there was no significant difference in age, pre-operative platelet count, histology, the diagnosis of deep vein thrombosis (DVT) and pulmonary thromboembolism, regimens of the first adjuvant chemotherapy, dose reduction during chemotherapy, or residual disease between the two groups. In terms of intervals from surgery to the day of blood test for each cycle of adjuvant chemotherapy, there were delays of 1–4 days in patients with splenectomy compared with patients without splenectomy during adjuvant chemotherapy.

**Table 1:** Baseline patients’ characteristics.

median (range or %)	Total (*n* = 474, 100 %)	No splenectomy(*n* = 395, 77.4 %)	Splenectomy(*n* = 79, 22.6%)	*p-value*
Age	54 (18-88)	54 (18-88)	55 (33-76)	0.322
Preoperative platelet counts *(x10^3^/mm^3^)*	308 (116-818)	305 (116-818)	325 (121-671)	0.128
Preoperative CA-125 *(IU/mL)*	617 (6-16719)	634 (6-15241)	859 (16-16719)	0.035
Number of cycles of adjuvant chemotherapy	6 (0-6)	6 (0-6)	6 (0-6)	0.182
FIGO Stage (*n, %)*				0.007
III	401 (84.6)	342 (86.6)	59 (74.7)	
IV	73 (15.4)	53 (13.4)	20 (25.3)	
Cell type (*n, %)*				0.055
Serous	405 (85.4)	332 (84.1)	73 (92.4)	
Non-serous	69 (14.6)	63 (15.9)	6 (7.6)	
Level of residual disease (*n, %)*				0.962
No gross residual	218 (46.0)	184 (46.6)	34 (43.0)	
1-9mm	108 (22.8)	85 (21.5)	23 (29.1)	
Equal to or more than 10 mm	148 (31.2)	126 (31.9)	22 (27.8)	
Deep vein thrombosis *(n, %)*	39 (8.2)	30 (6.3)	9 (1.9)	0.370
Pulmonary thromboembolism *(n, %)*	33 (7.0)	26 (5.5)	7 (1.5)	0.628
Regimens of 1^st^ adjuvant chemotherapy *(n, %)*				0.265
Paclitaxel-Carboplatin	400 (84.4)	336 (85.1)	64 (81.0)	
Paclitaxel-Carboplatin-Bevacizumab	62 (13.1)	50 (12.7)	12 (15.2)	
Docetaxel-Carboplatin	2 (0.4)	2 (0.5)	0 (0.0)	
Paclitaxel-Cisplatin	1 (0.2)	1 (0.3)	0 (0.0)	
Carboplatin	2 (0.4)	2 (0.5)	0 (0.0)	
FOLFOX-Bevacizumab	1 (0.2)	0 (0.0)	1 (1.3)	
none (included follow-up loss)	6 (1.3)	4 (1.0)	2 (2.5)	
Dose reduction on chemotherapy *(n, %)*	72 (15.2)	57 (14.4)	15 (19.0)	0.391
Interval between surgery and initiation of the first cycle of chemotherapy *(days, range)*	11 (6-61)	11 (6-55)	12 (7-61)	0.003
Interval from surgery to *(days, range)*				
Day of blood test^†^ for 1^st^ cycle Chemotherapy	9 (5-59)	8 (5-52)	10 (6-59)	0.001
Day of blood test^†^ for 2^nd^ cycle Chemotherapy	33 (26-86)	33 (26-81)	36 (28-86)	0.001
Day of blood test^†^ for 3^rd^ cycle Chemotherapy	57 (35-132)	56 (35-132)	60 (48-118)	0.002
Day of blood test^†^ for 4^th^ cycle Chemotherapy	80 (69-155)	80 (70-155)	82 (69-145)	0.006
Day of blood test^†^ for 5^th^ cycle Chemotherapy	104 (91-179)	104 (91-179)	106 (94-166)	0.021
Day of blood test^†^ for 6^th^ cycle Chemotherapy	127 (110-202)	127 (110-202)	128 (114-190)	0.188
Day of blood test^†^ after 6^th^ cycle chemotherapy	153 (133-224)	153 (133-224)	157 (133-211)	0.014

^†^Blood test contains complete blood cell count (CBC), chemistry, electrolyte, tumor marker.

CA-125, Cancer antigen 125; FOLFOX, folinic acid, fluorouracil, and oxaliplatin.

**Figure 1:**
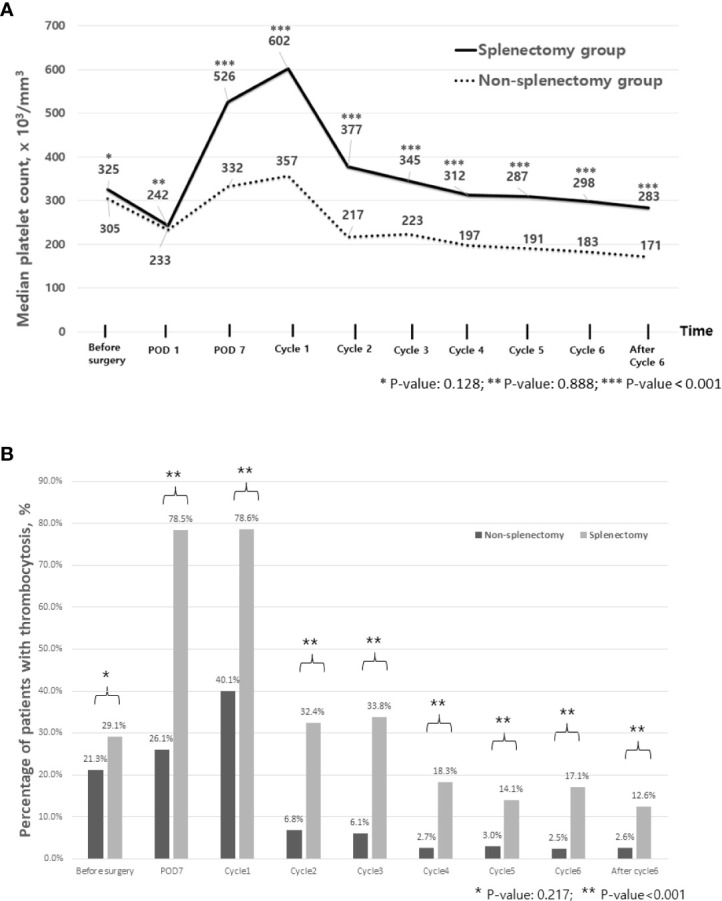
Trends of platelet counts and thrombocytosis during treatment. **(A)** Median platelet count during perioperative period and during adjuvant chemotherapy. **(B)** Percentage of patients with thrombocytosis during perioperative period and during adjuvant chemotherapy. Cycle 1 represents a day of blood test for the first cycle of adjuvant chemotherapy. POD, postoperative day.

Platelet counts were significantly increased after surgery. In the entire population, platelet counts were significantly elevated on POD 7 compared with pre-operative counts (344 × 10^3^/mm^3^ on POD 7 [95% CI 335–362, ×10^3^/mm^3^] *vs*. 308 × 10^3^/mm^3^ before surgery [296–318, ×10^3^/mm^3^], *p*-value <0.001). Additionally, the prevalence of thrombocytosis increased from 22.5% before surgery to 34.8% on POD 7. As shown in **Figure 1**, these findings were more frequently observed in patients with splenectomy. For example, the median platelet count on POD 7 was 526 × 10^3^/mm^3^ [95% CI 483–597, ×10^3^/mm^3^] in patients with splenectomy as opposed to 332 × 10^3^/mm^3^ [309–339, ×10^3^/mm^3^] in patients without splenectomy (*p*-value <0.001) and so does the prevalence of thrombocytosis on POD 7 (78.5% *vs*. 26.1%, *p*-value <0.001). Throughout the period of adjuvant chemotherapy, patients who underwent splenectomy showed significantly higher levels of platelet counts (*p*-value <0.001) and a higher prevalence of thrombocytosis (p <0.001) at each time point.

**Figure 2:**
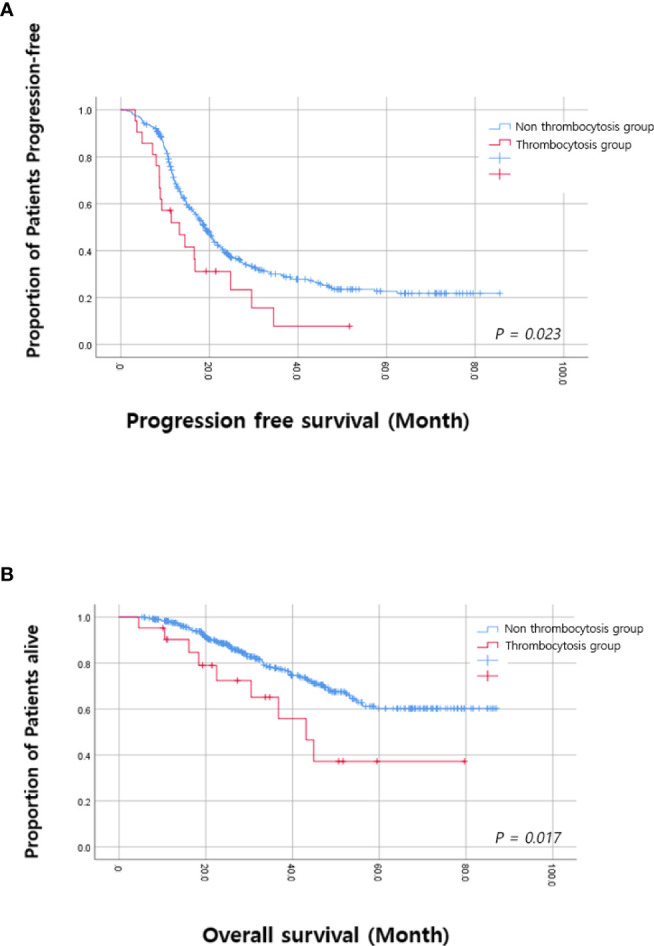
Kaplan–Meier analysis based on thrombocytosis at the fifth cycle **(A)** Progression free survival. **(B)** Overall survival.

Among the time points when platelet counts were available, we investigated to find the time point when thrombocytosis was associated with OS. We found that thrombocytosis on the day of the blood test for the fifth and sixth cycles of adjuvant chemotherapy was significantly associated with PFS and OS, as shown in **Table 2**. Therefore, we selected thrombocytosis on the day of the blood test for the fifth cycle of adjuvant chemotherapy as a representative marker for reactive thrombocytosis. In patients with thrombocytosis at this time point, 38.1% (8/21) showed persistent thrombocytosis at every time point during adjuvant chemotherapy. As shown in **Figure 2**, survival curves also demonstrated a significant difference based on thrombocytosis at the time of the fifth cycle of adjuvant chemotherapy. Median progression-free survival was 12.1 months (95%CI 5.6–20.5 months) in patients with thrombocytosis and 19.0 months (95%CI 17.2–20.8 months) in patients without thrombocytosis (*p*-value = 0.001). In terms of OS, the survival rate at 3 years was 62.6% in patients with thrombocytosis and 78.8% in patients without thrombocytosis.

**Table 2:** Univariate analysis: effect of platelet count at each time point on PFS and OS.

		PFS		OS	
		HR (95% CI)	*p*-value	HR (95% CI)	*p*-value
Before surgery	Without thrombocytosis (N=367)	1 (reference)		1 (reference)	
	With thrombocytosis (N=107)	1.400(1.075-1.822)	0.012	1.479(0.986-2.220)	0.059
POD 1	Without thrombocytosis (N=449)	1 (reference)		1 (reference)	
	With thrombocytosis (N=25)	2.030(1.300-3.170)	0.002	0.825(0.337-2.021)	0.674
POD 7	Without thrombocytosis (N=309)	1 (reference)		1 (reference)	
	With thrombocytosis (N=165)	1.548(1.228-1.953)	<0.001	1.321(0.916-1.906)	0.135
1^st^ Cycle of chemotherapy	Without thrombocytosis (N=249)	1 (reference)		1 (reference)	
	With thrombocytosis (N=215)	1.325(1.054-1.664)	0.016	1.016(0.705-1.466)	0.931
2^nd^ Cycle	Without thrombocytosis (N=402)	1 (reference)		1 (reference)	
	With thrombocytosis (N=50)	1.737(1.246-2.421)	0.001	1.361(0.802-2.310)	0.252
3^rd^ Cycle	Without thrombocytosis (N=396)	1 (reference)		1 (reference)	
	With thrombocytosis (N=47)	1.736(1.236-2.438)	0.001	1.455(0.843-2.510)	0.178
4^th^ Cycle	Without thrombocytosis (N=417)	1 (reference)		1 (reference)	
	With thrombocytosis (N=23)	1.285(0.775-2.129)	0.330	1.861(0.904-3.829)	0.087
5^th^ Cycle	Without thrombocytosis (N=416)	1 (reference)		1 (reference)	
	With thrombocytosis (N=21)	2.012(1.231-3.290)	0.005	2.370(1.152-4.875)	0.019
6^th^ Cycle	Without thrombocytosis (N=416)	1 (reference)		1 (reference)	
	With thrombocytosis (N=21)	2.255(1.413-3.600)	0.001	1.595(0.741-3.435)	0.229
After 6^th^ cycle	Without thrombocytosis (N=404)	1 (reference)		1 (reference)	
	With thrombocytosis (N=18)	1.546(0.903-2.647)	0.112	1.845(0.856-3.976)	0.118

Thrombocytosis was defined as platelet count ≥ 4.0 x 10^5^/mm^3^.

PFS, progression free survival; OS, overall survival; HR, hazard ratio; CI, confidential interval; POD, postoperative day. Every cycle represents the day for blood test for each cycle of adjuvant chemotherapy.

Multivariate analysis adjusting for pre-operative platelet counts, age, FIGO stage, intervals between surgery and the first cycle of adjuvant chemotherapy, and residual diseases, etc. was performed and we found that thrombocytosis on the day of blood test for the fifth cycle of adjuvant chemotherapy was an independent prognostic factor for poor PFS (HR; 1.894, 95% CI; 1.157–3.101, *p*-value = 0.011) and OS (HR; 2.483, 95% CI; 1.205–5.117, *p*-value = 0.014) as shown in **Tables 3** and**4**. Additionally, levels of residual disease and intervals between surgery and the initiation of the first cycle of chemotherapy were also significant prognostic factors for OS. Of note, splenectomy did not affect PFS (*p*-value = 0.180) or OS (*p*-value = 0.947).

**Table 3:** Univariate and multivariate analysis for progression free survival.

	Univariate		Multivariate	
	HR (95% CI)	*p*-value	HR (95% CI)	*p*-value
Age	1.002 (0.991-1.013)	0.739	0.993 (0.981-1.005)	0.311
Platelet count, before surgery				
Without thrombocytosis	1 (reference)		1 (reference)	
With thrombocytosis	1.400 (1.075-1.822)	0.012	1.001 (1.000-1.002)	0.114
Platelet count, 5^th^ cycle				
Without thrombocytosis	1 (reference)		1 (reference)	
With thrombocytosis	2.012 (1.231-3.290)	0.005	1.894 (1.157-3.101)	0.011
TTC, days	1.001 (0.995-1.007)	0.701	1.002 (0.995-1.008)	0.755
Stage				
III	1 (reference)		1 (reference)	
IV	1.444 (1.068-1.952)	0.017	1.144 (0.832-1.572)	0.371
CA-125	1.000 (1.000-1.000)	0.006	1.000 (1.000-1.000)	0.089
Deep vein thrombosis	1.592 (1.091-2.321)	0.016	1.359 (0.868-2.126)	0.179
Pulmonary thromboembolism	2.078 (1.415-3.052)	<0.001	1.903 (1.252-2.891)	0.003
Dose reduction on chemotherapy	1.399 (1.048-1.868)	0.023	1.266 (0.909-1.762)	0.162
Regimens of 1^st^ adjuvant chemotherapy				
TC	1 (reference)		1 (reference)	
TC + Bevacizumab	1.223 (0.863-1.732)	0.258	0.401 (0.254-0.633)	<0.001
Others	1.614 (0.761-3.425)	0.212	5.401 (1.616-18.054)	0.006
Number of cycles of 1^st^ adjuvant chemotherapy				
6 to 9	1 (reference)		1 (reference)	
1 to 5	1.732 (0.970-3.092)	0.063	17.691 (3.889-80.480)	<0.001
Splenectomy				
Without splenectomy	1 (reference)		1 (reference)	
With splenectomy	1.419 (1.072-1.877)	0.014	1.179 (0.863-1.612)	0.180
Level of residual disease (N, %)				
No gross residual	1 (reference)		1 (reference)	
1-9mm	1.750 (1.310-2.337)	<0.001	1.705 (1.269-2.292)	<0.001
Equal to or more than 10mm	2.162 (1.658-2.819)	<0.001	2.056 (1.569-2.695)	<0.001

Thrombocytosis was defined as platelet count ≥ 4.0 x 10^5^/mm^3^.

HR hazard ratio, CI confidential interval, CA-125 Cancer antigen 125, TTC time from surgery to the first cycle of chemotherapy, TC paclitaxel-carboplatin.

**Table 4:** Univariate and multivariate analysis for overall survival.

	Univariate		Multivariate	
	HR (95% CI)	*p*-value	HR (95% CI)	*p*-value
Age	1.026 (1.008-1.044)	0.004	1.013 (0.993-1.033)	0.221
Platelet count, before surgery				
Without thrombocytosis	1 (reference)		1 (reference)	
With thrombocytosis	1.479 (0.986-2.220)	0.059	1.002 (1.000-1.003)	0.099
Platelet count, 5^th^ cycle				
Without thrombocytosis	1 (reference)		1 (reference)	
With thrombocytosis	2.370 (1.152-4.875)	0.019	2.483 (1.205-5.117)	0.014
TTC, days	1.011 (1.005-1.016)	<0.001	1.010 (1.004-1.016)	0.001
Stage				
III	1 (reference)		1 (reference)	
IV	1.267 (0.775-2.072)	0.344	0.912 (0.530-1.570)	0.677
CA-125	1.000 (1.000-1.000)	0.417	1.000 (1.000-1.000)	0.814
Deep vein thrombosis	1.619 (0.940-2.786)	0.082	1.169 (0.590-2.318)	0.654
Pulmonary thromboembolism	1.857 (1.022-3.374)	0.042	1.773 (0.921-3.414)	0.087
Dose reduction on chemotherapy	1.775 (1.184-2.663)	0.006	1.377 (0.841-2.257)	0.204
Regimens of 1^st^ adjuvant chemotherapy				
TC	1 (reference)		1 (reference)	
TC + Bevacizumab	1.131 (0.586-2.184)	0.714	0.942 (0.455-1.952)	0.872
Others	1.942 (0.715-5.275)	0.193	2.564 (0.272-24.207)	0.411
Number of cycles of 1^st^ adjuvant chemotherapy				
6 to 9	1 (reference)		1 (reference)	
1 to 5	4.893 (2.684-8.920)	<0.001	0.000 (0.000-)	0.967
Splenectomy				
Without splenectomy	1 (reference)		1 (reference)	
With splenectomy	1.279 (0.837-1.954)	0.254	0.982 (0.599-1.609)	0.947
Level of residual disease (N, %)				
No gross residual	1 (reference)		1 (reference)	
1-9 mm	2.259 (1.408-3.624)	0.001	2.480 (1.512-4.068)	<0.001
Equal to or more than 10 mm	2.790 (1.805-4.311)	<0.001	2.901 (1.830-4.598)	<0.001

Thrombocytosis was defined as platelet count ≥ 4.0 x 10^5^/mm^3^.

HR, hazard ratio; CI, confidential interval; CA-125, Cancer antigen 125; TTC, time from surgery to the first cycle of chemotherapy; TC, paclitaxel-carboplatin.

A logistic regression model was used to find clinical factors attributing thrombocytosis and splenectomy (*p*-value <0.001) was the independent factor associated with thrombocytosis on the day of the blood test for the fifth cycle of adjuvant chemotherapy shown in **Supplementary Table 1**. Thromboembolic events and overwhelming post-splenectomy infection syndrome (OPIS) were not observed during the study period.

## Discussion

To the best of our knowledge, it is the first study investigating the incidence of reactive thrombocytosis after primary cytoreductive surgery in advanced ovarian cancer and its role in survival in such a large cohort. Previous studies demonstrated the pre-treatment platelet count as a prognostic factor for survival (22–24). However, none of these studies reported the role of thrombocytosis after surgery. At the same time, the incidence of reactive thrombocytosis after splenectomy, which is a procedure during primary cytoreductive surgery frequently performed to achieve optimal cytoreduction (25) and how long it lasts in advanced ovarian cancer, has not been reported.

We found that reactive thrombocytosis after primary cytoreductive surgery occurred in approximately 40% of patients without splenectomy and 80% of patients with splenectomy during the perioperative period, and 10%–20% of patients with splenectomy showed persistent thrombocytosis until the end of adjuvant chemotherapy, which is significantly higher compared with approximately 2% in patients without splenectomy. Compared with patients with normal platelet counts after surgery, patients who showed persistent thrombocytosis after primary cytoreductive surgery showed inferior survival, suggesting that new-treatment strategies, including antiplatelet therapy or platelet-mediated signaling pathway blockers, should be considered in these patients (16).

Physiologically, platelets are responsible for hemostasis, immunity, and inflammation (26). However, in malignancy, evidence suggests that platelets play a role in tumor growth and metastasis (27, 28). For example, pre-clinical studies found that activated platelets stimulate angiogenesis by releasing the content of their granules, containing numerous growth factors such as platelet-derived growth factor (PDGF) and vascular endothelial growth factors (VEGF) (29). Platelets protect cancer cells from immune surveillance and facilitate hematogenic tumor spread by forming tumor cell–platelet aggregates in capillary beds (15). The association of pre-treatment thrombocytosis with poor prognosis has also been described in patients with solid malignancies (8, 11–14). In EOC, most studies have shown that thrombocytosis at initial diagnosis is associated with a shorter PFS or OS (16, 30–35). One of the possible mechanisms explaining pre-operative thrombocytosis in patients with EOC is an activated paracrine signaling pathway. For example, interleukin-6 (IL-6) released from ovarian cancer cells can stimulate the secretion of thrombopoietin in the liver and it eventually leads to thrombocytosis. Then, tumor progression and metastasis can be enhanced by thrombocytosis and, in the end, more IL-6 will be released from these tumors, forming a vicious circle (16, 36). This is supported by the evidence that silencing IL-6 and thrombopoietin abrogates thrombocytosis in animal models (16), and pre-treatment thrombocytosis in patients with EOC tends to be related to advanced stage, higher grade, higher level of CA-125, larger ascites volume, and larger tumor residual (31–35).

Aside from thrombocytosis as a response to neoplasms as mentioned above, various conditions such as major trauma (37) and surgeries have also been known to cause thrombocytosis. In one study, the overall incidence of thrombocytosis was 18.7% in patients who were admitted to the intensive care unit (ICU) for trauma (38) and it was associated with higher rates of complications, particularly venous thromboembolism. Additionally, thrombocytosis was associated with the severity of an injury (39). In another study, persistent thrombocytosis in critically injured patients receiving routine chemoprophylaxis was associated with thrombotic complications (40), suggesting that persistent thrombocytosis may be more critical in relation to poor outcomes. There are several reports investigating the effects of thrombocytosis after surgery in solid tumors. Some suggest the elevation of platelet count after surgery is associated with post-operative complications (6, 41). For example, 37% of patients who underwent colorectal surgery developed post-operative thrombocytosis (defined as platelets ≥5.0 × 10^5^/mm^3^) with a peak on the 8th day after surgery (a range of 1–49 days) and a positive correlation between post-operative thrombocytosis and complications was found (6). Another study looked at patients who received urologic surgery. Approximately 90% of patients with post-operative thrombocytosis (defined as platelets ≥5.0 × 10^5^/mm^3^) were diagnosed with post-operative complications such as urosepsis, hemorrhage, and thromboembolism (41).

In relation to platelet counts at various time points and prognosis in ovarian cancer, there was a report showing that lower platelet counts before chemotherapy were associated with better survival and higher platelet counts were more frequent in patients who had recurrent disease as compared with patients without recurrence (36). However, reactive thrombocytosis after maximal cytoreductive surgery was not assessed in this study. More on that, platelet counts were significantly lower at post-operative time point compared with baseline, and on which post-operative day the blood sampling was done is unclear. Another study evaluated the platelet to lymphocyte ratio (PLR) in ovarian cancer and showed that a high PLR of more than 225 was associated with a higher risk of progression and shorter survival than a lower PLR (42). Since high platelet count after surgery may reflect high tumor volume or high level of surgical complexity (43), frailty of patients could also be a possible reason explaining the association between thrombocytosis and poor survivals (44–46). It is unknown that advanced ovarian cancer patients with enhanced recovery after surgery (ERAS) protocol (47) are associated with better survival by alleviating surgical stress, and it is worth investigating in the future.

There is very limited evidence investigating the impact of post-operative thrombocytosis on survival outcomes and one study showed that post-operative thrombocytosis (11.9% of patients under the definition of platelets >4.0 × 10^5^/mm^3^) was one of the significant independent prognostic markers for poor survival in patients with colorectal cancers (in a multivariate analysis, HR; 1.98, 95% CI; 1.12–3.49, *p*-value = 0.018) (18). In our study, 4.82% in all study population (14.08% in patients with splenectomy and 3.79% in patients without splenectomy) showed post-operative thrombocytosis (defined as platelets ≥4.0 × 10^5^/mm^3^) on the fifth cycle of chemotherapy approximately 3 months after surgery when it has the most significant impact on survival and the HR for OS was 2.483 (95% CI; 1.205–5.117, *p*-value = 0.014) which is corresponding well with that of the above study. Considering that reactive thrombocytosis after splenectomy for non-malignant disease persisted for one year (10), platelet counts in our study, specifically of patients with splenectomy, might have been undermeasured due to bone marrow suppression from chemotherapy.

Since the definition of thrombocytosis, the timing of blood tests showing thrombocytosis, and the number of cycles of adjuvant chemotherapy that patients received should be specific to our cohort, it is difficult to extrapolate our results to general populations. For example, the definition of thrombocytosis varies according to previous studies (11, 12, 20, 21). Accordingly, we defined thrombocytosis as a platelet count ≥4.0 x 10^5^/mm^3^. Also, as opposed to patients who had suboptimal cytoreduction, patients with optimal cytoreduction were highly likely to show persistent thrombocytosis in our analysis. There is a report showing a greater rise in platelet counts in the caesarean section group compared with the vaginal delivery group (48) in pregnant women, suggesting a positive correlation between levels of surgical trauma and the severity of thrombocytosis. However, it is still unclear whether the surgical complexity (e.g., surgical extent, multiple procedures, etc.) in surgical patients is associated with the severity of surgery-induced thrombocytosis. On the other hand, reactive thrombocytosis is one of the well-known complications of splenectomy (49). Because old platelets are destroyed by phagocytosis in the spleen after circulating for 7–11 days in the blood, about 75% of individuals without myeloproliferative disorders develop thrombocytosis after splenectomy in the general population (9). In our study, 78.5% of patients who had splenectomy showed thrombocytosis approximately 7–9 days after surgery, which led us to consider splenectomy as one of the confounders in our study. In multivariate analyses, we found splenectomy itself was not associated with poor survival but was one of the main contributors to persistent thrombocytosis after surgery. We should be careful to interpret these findings since suboptimal cytoreduction is still an independent prognostic factor for poor survival in our study, suggesting that advantages from removing tumors on the spleen to achieve optimal cytoreduction may outweigh disadvantages from splenectomy induced thrombocytosis. From our results, we cannot state that transfusion of platelets should be avoided even when bone marrow suppression is critical during chemotherapy. Nevertheless, we need new strategies to increase oncological outcomes in subgroup of patients with advanced EOC, especially who had persistent thrombocytosis after splenectomy during primary cytoreductive surgery.

Apart from the retrospective study design, there are more limitations in our study. We did not provide any role for platelet count in predicting complications after surgery (50, 51) as suggested by previous studies, which may be useful in communication about post-operative course and when to start adjuvant chemotherapy with patients and their caregivers. However, as of our knowledge, this is the first article demonstrating the relationship between persistent thrombocytosis after primary cytoreductive surgery and oncologic outcomes as previous studies have described that thrombocytosis at initial diagnosis is associated with negative oncologic outcome in EOC (16, 30–35).

In conclusion, reactive thrombocytosis after primary cytoreductive surgery is frequently observed during adjuvant chemotherapy among women with advanced ovarian cancer. Regardless of the presence or absence of preoperative thrombocytosis, which is known as a prognostic factor from the previous literature, the role of reactive thrombocytosis after primary cytoreductive surgery on oncological outcomes has not been elucidated and we found that it was independently associated with poor survival. In particular, patients who had splenectomy during primary cytoreductive surgery showed a higher incidence of reactive thrombocytosis. These findings do not mean that avoiding splenectomy is excused if there is a chance of achieving optimal cytoreduction with splenectomy. Conversely, we need more research to circumvent inferior survival from thrombocytosis induced by maximal cytoreductive surgery in advanced ovarian cancer as a next step.

## Data availability statement

The raw data supporting the conclusions of this article will be made available by the authors, without undue reservation.

## Ethics statement

The studies involving human participants were reviewed and approved by the Institutional Review Board (IRB) of Samsung Medical Center. Written informed consent for participation was not required for this study in accordance with the national legislation and the institutional requirements.

## Author contributions

Y-YL: Conceptualization, funding acquisition, methodology, supervision, and writing—review and editing. M-SK: Supervision, statistical analysis, and writing—review and editing. SB: Data collection, statistical analysis, and writing—original draft. JN: Data collection. JS: Data collection. JK: Data collection. SJ: Data collection. CC: Methodology. T-JK: Methodology. J-WL: Methodology. All authors contributed to the article and approved the submitted version.

## Funding

This work was supported by the National Research Foundation of Korea (NRF) grant funded by the Korea government (MSIT) (2019R1F1A1063567). This research was supported by Research and Business Development Program through the Korea Institute for Advancement of Technology (KIAT) funded by the Ministry of Trade, Industry and Energy (MOTIE) (P0014051).

## Conflict of interest

The authors declare that the research was conducted in the absence of any commercial or financial relationships that could be construed as a potential conflict of interest.

## Publisher’s note

All claims expressed in this article are solely those of the authors and do not necessarily represent those of their affiliated organizations, or those of the publisher, the editors and the reviewers. Any product that may be evaluated in this article, or claim that may be made by its manufacturer, is not guaranteed or endorsed by the publisher.
