# Supplementary material for: Role of reactive thrombocytosis after primary cytoreductive surgery in advanced ovarian cancer
Source: Front Oncol. 2022 Sep 8;12:926878. doi: 10.3389/fonc.2022.926878 (PMC9493080; doi:10.3389/fonc.2022.926878)
Supplement: Supplementary file 1 [file Table_1.docx]

**Supplementary table 1.** Logistic regression analysis of attributing factors for the thrombocytosis at 5^th^ cycle

|  | | B | S.E. | Wald | df | Sig. | Exp(B) | 95% C.I, for EXP(B) | |
| --- | --- | --- | --- | --- | --- | --- | --- | --- | --- |
|  |  |  |  |  |  |  |  | Lower | Upper |
| 1^st^ cycle of chemotherapy | Age | -0.024 | 0.024 | 1.011 | 1 | 0.315 | 0.976 | 0.931 | 1.023 |
|  | Thrombocytosis, before surgery | -1.150 | 0.505 | 5.175 | 1 | 0.023 | 0.317 | 0.118 | 0.853 |
|  | TTC | -0.003 | 0.022 | 0.022 | 1 | 0.882 | 0.997 | 0.954 | 1.041 |
|  | Stage | -0.687 | 0.528 | 1.697 | 1 | 0.193 | 0.503 | 0.179 | 1.414 |
|  | CA-125 | 0.000 | 0.000 | 1.851 | 1 | 0.174 | 1.000 | 1.000 | 1.000 |
|  | Splenectomy | 2.176 | 0.499 | 19.005 | 1 | 0.000 | 8.807 | 3.312 | 23.421 |
|  | No gross residual |  |  | 3.361 | 2 | 0.186 |  |  |  |
|  | 1-9 mm | -0.285 | 0.695 | 0.168 | 1 | 0.682 | 0.752 | 0.192 | 2.937 |
|  | Equal to or mor than 10 mm | 0.809 | 0.575 | 1.980 | 1 | 0.159 | 2.245 | 0.728 | 6.925 |
|  | Constant | -1.085 | 1.371 | 0.626 | 1 | 0.429 | 0.338 |  |  |
| 5^th^ cycle | Thrombocytosis, before surgery | -1.058 | 0.471 | 5.038 | 1 | 0.025 | 0.347 | 0.138 | 0.874 |
|  | Splenectomy | 2.005 | 0.469 | 18.312 | 1 | 0.000 | 7.428 | 2.965 | 18.610 |
|  | Constant | -2.949 | 0.436 | 45.771 | 1 | 0.000 | 0.052 |  |  |

Thrombocytosis was defined as platelet count ≥ 4.0 x 10^5^/mm^3^

*B* beta coefficients, *S.E* standard errors, *Wald* wald statistic, *Df* degress of freedom, *Sig* significance level, *Exp(B)* exponentiation of the beta coefficients, *CI* confidential interval, *CA-125* Cancer antigen 125, *TTC* Time from surgery to the first cycle of chemotherapy
